# Selection and Yield Formation Characteristics of Dry Direct Seeding Rice in Northeast China

**DOI:** 10.3390/plants12193496

**Published:** 2023-10-07

**Authors:** Chao Liang, Yimeng Li, Kunhao Zhang, Zhouzhou Wu, Jiaxin Liu, Junfeng Liu, Chanchan Zhou, Shu Wang, Fenghai Li, Guomin Sui

**Affiliations:** 1 College of Agronomy, Shenyang Agricultural University, Shenyang 110866, China; liangchao@stu.syau.edu.cn (C.L.); liym2019@stu.syau.edu.cn (Y.L.); 2021240344@stu.syau.edu.cn (K.Z.); 2022200082@stu.syau.edu.cn (Z.W.); 2020220276@stu.syau.edu.cn (J.L.); junfengliu58@163.com (J.L.); lifenghai@126.com (F.L.); 2 Liaoning Academy of Agricultural Sciences, Shenyang 110161, China; guomin666@126.com

**Keywords:** dry direct seeding rice, cultivar selection, grain yield, yield level

## Abstract

Dry direct seeding rice (DSR) is an emerging production system because of increasing labor and water scarcity in rice cultivation. The limited availability of rice cultivars suitable for dry direct seeding hampers the widespread adoption of this cultivation method in Northeast China. This study aimed to investigate grain production and plant characteristics associated with dry direct seeding rice. We conducted a field experiment on 79 *japonica* rice cultivars in Shenyang City, Liaoning Province, Northeast China, in 2020 and 2021. This study found that the grain yield of the tested rice cultivars ranged from 5.75–11.00 t ha^−1^, with a growth duration lasting between 144–161 days across the cultivars. These cultivars were then categorized into high yielding (HY), medium yielding (MY), and low yielding (LY) based on daily yield by using Ward’s hierarchical clustering method. The higher grain yield for HY compared to MY and LY was attributed to more spikelets per unit area. The HY alleviated the conflict between higher panicle density and larger panicle size by improving the seedling emergence rate and productive stem rate. It also significantly increased shoot biomass at maturity. The HY reduced the period between seeding and beginning of heading (BBCH 51) and the proportion of dry matter partitioned to the leaf at the heading stage. However, it also increased the accumulation of dry matter in the grain and the proportion of dry matter partitioned to the grain at maturity. Furthermore, the HY markedly increased the harvest index and grain-leaf ratio, which are beneficial to coordinate the source–sink relationship. A quadratic function predicted that 98 days is the optimum growth duration before heading (BBCH 51) for achieving maximum yield. In conclusion, for dry direct seeding rice, it is appropriate to select high-yielding *japonica* inbred rice cultivars with shorter growth duration before heading (about 93–102 day), higher panicle number (about 450–500 × 10^4^ ha^–1^), more spikelet number per panicle (about 110–130), higher seedling emergence rate (about 70–75%), higher productive stem rate (about 60–70%), and greater harvest index (about 50–55%).

## 1. Introduction

Rice (*Oryza sativa* L.) is the primary crop and constitutes a staple diet for over half of the global population [1]. Puddled transplanted rice (PTR) is the primary method for rice establishment in the majority of East Asia. In China, 95% of the rice is cultivated under puddled transplanted conditions that involve prolonged periods of flooding [2]. However, sustainable production of puddled transplanted rice has been severely limited due to various issues, such as labor shortage and water scarcity [3]. Puddle-transplanted rice uses large amounts of irrigation or rainfall water during land preparation and growth [4], with much lost to evaporation and percolation, resulting in poor water productivity [5]. Furthermore, conventional rice transplanting is labor intensive since puddled-transplanted rice necessitates puddling the rice field, raising a nursery for the seedlings and transplanting them [6]. Despite this, urbanization and the migration of workers from the agricultural sector to non-agricultural and industrial sectors have limited the availability of labor, leading to higher costs of rice production [7]. Considering the drawbacks of puddled-transplanted rice, there is a pressing need for less labor-intensive and more cost-effective methods of rice production to promote sustainable cultivation practices [8,9,10].

Direct seeding technology is seen as a promising solution to the problem of insufficient water and labor resources for sustainable rice production, with the aims of saving water and labor and increasing productivity in the system [11,12]. Dry direct seeding rice refers to the process of establishing the crop from seeds sown in the nonpuddled and unsaturated soil. In contrast, the seedlings from nursery are transplanted in the puddle soil in transplanted-flooded rice [13]. Dry direct seeding rice requires 30–51% less total water for land preparation and is dependent on soil types, providing 32–88% higher crop productivity and saving 49–55% on labor compared to puddled transplanted rice [14]. Dry direct seeding rice often associated with lower yields than transplanted rice, although it does address some of the problems associated with water and labor shortages [6,15,16]. The causes of lower yields of dry direct seeding rice are variable and include poor crop establishment [17], inadequate weed control [18], panicle sterility [19], lodging [20], and lacks of suitable varieties [21] and agronomic practices [22]. Among which, the lack of stable yielding and adapted varieties for dry direct seeding system is the major limitation in achieving the maximum yield potential [23]. To overcome this obstacle, developing rice varieties with high yield potential is one possible solution.

Currently, the cultivars being used in the dry direct seeding system are those bred for puddled-transplanted rice; these cultivars are inadequate, as they fail to realize their full yield potential when grown under a dry direct seeding system [24]. Since the 1970s, considerable research has been conducted to explore appropriate rice cultivars, crop management, and weed control measures for dry direct seeding rice [25,26,27]. Nevertheless, there are limited published reports on the yield performance of dry direct seeding rice in Northeast China. To breed high-yielding varieties for dry direct seeding rice in North East China, it is necessary to comprehend the conditions of grain development and crop dynamics of diverse cultivars (originally bred for puddled transplanted rice) within the dry direct seeding system and assess their yield potential. Dry direct seeding rice is usually sown at early May in Northeast China. Nevertheless, the low temperature after sowing severely hampers the seed germination and seedling growth [28], which may prolong the growth duration of local rice varieties. In order to optimize rice production in Northeast China, where the timing is constrained by light and temperature resources, cultivars with a short growth duration should be chosen for dry direct seeding. Furthermore, the productivity of dry direct seeding rice is also limited by the sink size [29]. Determining the suitable growth duration for dry direct seeding varieties is a benefit to improved assimilation and nutrient uptake and fills physiological gaps that limit the sink size and productivity in dry direct seeding systems [30]. Thus, it is imperative to consider both grain yield and growth duration while choosing rice cultivars suitable for dry direct seeding. Farmers still struggle to properly evaluate the importance of both growth duration and grain yield at the same time. It is necessary to establish straightforward selection criteria for sustainable cultivars for dry direct seeding rice cultivation, especially in the *japonica* inbred rice region located in Northeast China, where the availability of light and temperature is limited. Daily yield per unit area, which takes into account both grain yield and growth duration, has been advocated as a vital criterion for identifying the adaptability of rice cultivars for puddled transplanting [31,32]. The higher the daily yield of rice cultivars, the greater the adaptability to puddled transplanting [7,33]. However, the information is limited about the growth and yield formation characteristics of high daily yield dry direct seeding rice cultivars.

Therefore, a field experiment was carried out in Shenyang City, Liaoning Province, China in 2020 and 2021. This study categorized 79 *japonica* rice cultivars into three types based on daily yield through hierarchical cluster analysis. The objectives of this study were: (1) to evaluate the yield and yield components of dry direct seeding rice of the three different cultivar types; (2) to measure the production of dry matter, distribution, and source–sink characteristics of the different cultivar types; and (3) to identify the relationships among growth duration and grain yield. The results of this study might offer both theoretical and empirical foundations for the selection and breeding of rice cultivars with high-yielding that are appropriate for dry direct seeding systems.

## 2. Results

### 2.1. Grain Yield, Daily Yield and Yield Components

**Figure 1:**
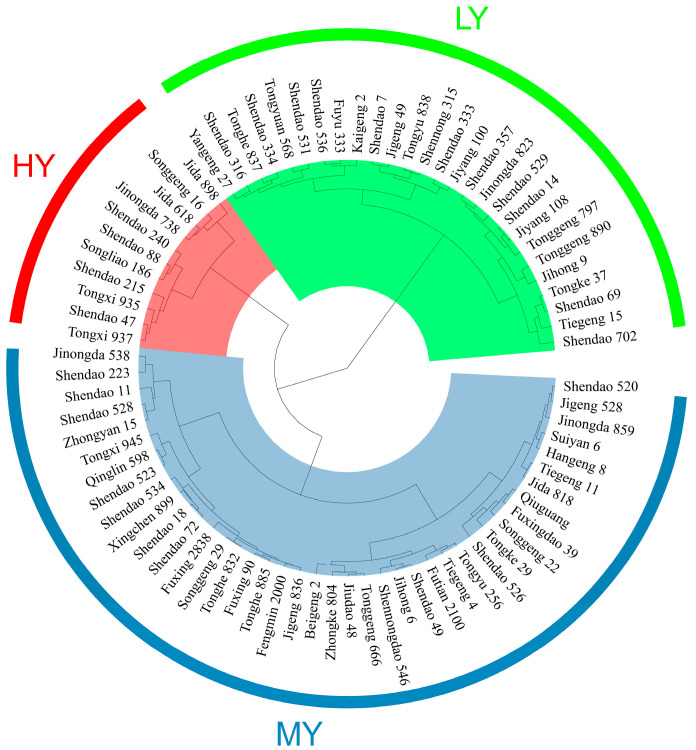
Hierarchical cluster analysis of grain yield of 79 dry direct seeding rice cultivars. HY, high-yielding cultivars; MY, medium-yielding cultivars; LY, low-yielding cultivars.

The grain yield, daily yield, and whole growth duration of 79 selected cultivars ranged from 5.75–11.00 t ha^−1^, 27.48–70.98 kg ha^–1^ d^–1^, and 144–161 d. The selected rice cultivars were separated into three cultivar types, including high yielding (HY), medium yielding (MY), and low yielding (LY), using Ward’s method for hierarchical clustering (Figure 1).

Compared with MY and LY, HY had 23.0% and 42.8% higher grain yield and 22.5% and 42.5% higher daily yield, respectively. Table 1 shows that there were no significant differences in the grain filling percentage among the three cultivar types. However, the number of effective panicles per m^2^, number of spikelets per unit area, number of spikelets per panicle, 1000-grain weight, and grain yield were significantly influenced by the cultivar type. The 1000-grain weight was in the order of LY higher than HY higher than MY. Compared with MY and LY, the number of spikelets m^−2^ of HY were 17.6% and 38.3% higher, respectively, which was owing to the high number of effective panicles and spikelets per panicle. Compared with MY and LY, the number of effective panicles m^−2^ of HY were 9.5% and 32.9% higher, respectively; spikelets per panicle of HY were 8.5% and 8.3% higher than those of MY and LY, respectively. Additionally, there was no difference in the grain filling rate among HY, MY, and LY.

### 2.2. Growth Duration, Seedling Emergence Rate, Number of Stems, and Productive Stem Rate

**Figure 2:**
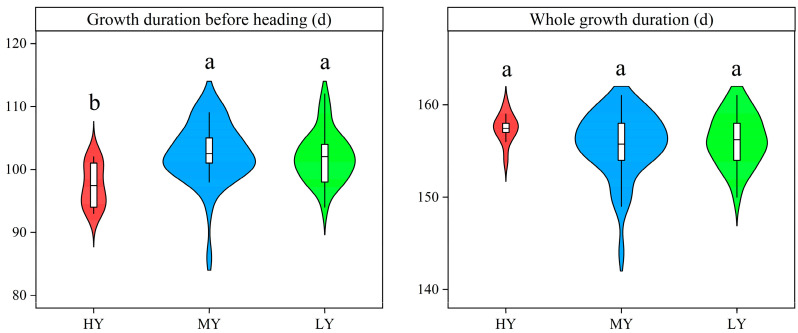
Growth durations for the three dry direct seeding rice types of cultivars. Each plot displays the interquartile range, with the mean marked by a horizontal line within the box. Cultivars with significant differences (*p* < 0.05) are identified by different lowercase letters. HY, MY, and LY refer to high-yielding cultivars, medium-yielding cultivars, and low-yielding cultivars, respectively. The same definitions are used as below.

**Figure 3:**
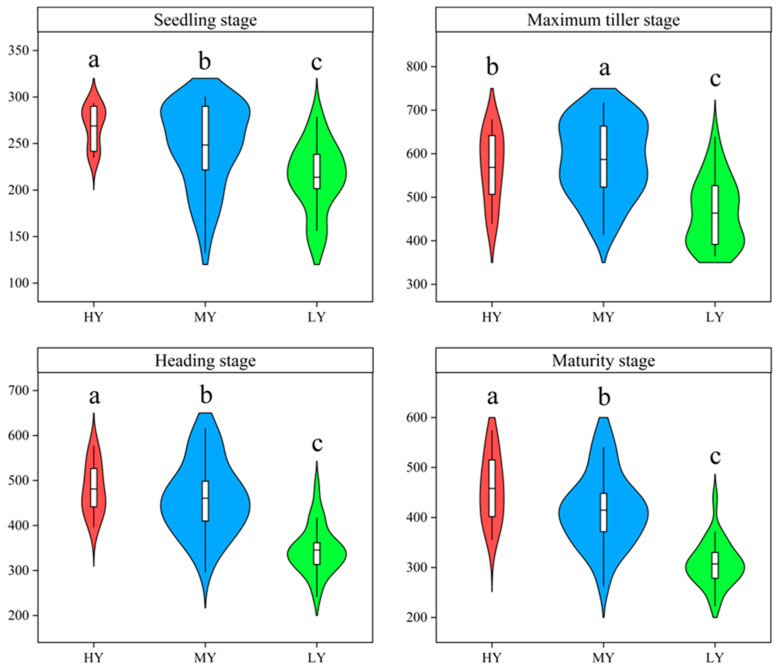
Tiller and panicle numbers per square meter of different cultivars during each growth stage. Cultivars with significant differences (*p* < 0.05) are identified by different lowercase letters.

**Figure 4:**
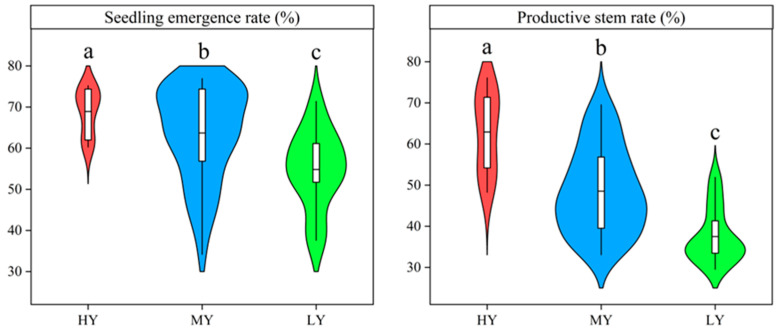
Seedling emergence rate and productive stem rate of different cultivars. Cultivars with significant differences (*p* < 0.05) are identified by different lowercase letters.

MY and LY did not markedly differ in the growth duration before heading (BBCH 51) (Figure 2). HY had shorter growth durations before heading (BBCH 51). Hence, the growth durations before heading (BBCH 51) were 5.06 and 4.58 d shorter than those for MY and LY, respectively. The number of stems or panicles were markedly affected by cultivar types at each stage, excluding maximum tillering stage (Figure 3). HY and MY had a greater number of stems or panicles. The stems or panicles of HY and MY increased 16.19–25.74%, 22.68–26.57%, 33.39–39.35%, and 34.99–49.12% more than that for LY at seedling stage, maximum tillering stage, heading stage, and maturity stage, respectively. The seedling emergence rate and productive stem rate differed significantly among cultivars (Figure 4), and the orders were both HY > MY > LY. Compared to MY and LY, seedling emergence rate of HY increased by 8.22% and 25.74%, respectively, which explained that the HY had more seedlings at seedling stage. HY and MY did not markedly differ in the number of stems at maximum tillering stage. However, productive stem rate of HY increased by 29.60%, resulting in a significantly higher panicle number relative to MY at heading stage. Thus, HY presented comparatively greater seedling emergence ability and higher productive stems.

### 2.3. Dry Matter Accumulation and Allocation

**Figure 5:**
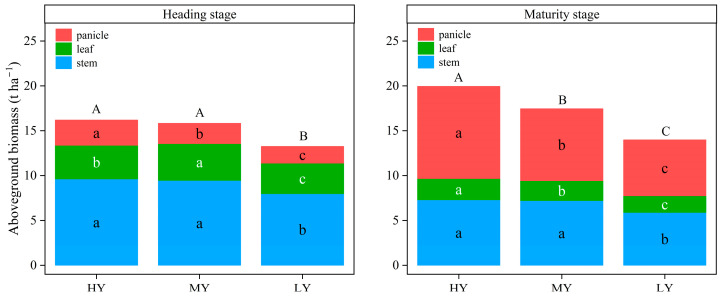
Aboveground biomass for the three dry direct seeding rice cultivars at heading stage and maturity stage. For a given plant organ, different lowercase letters among columns indicate significant (*p* < 0.05) differences in dry matter accumulation amount among cultivars; different capital letters within a column indicate significant (*p* < 0.05) differences in biomass among cultivars.

Cultivar type markedly affected stem, leaf, and panicle biomass amount at each growth stage (Figure 5). HY and MY had significantly greater dry matter accumulation amount at each stage compared to LY. Compared with LY, HY and MY had 22.22% and 19.43% more dry matter accumulation at heading stage, respectively, and 42.64% and 24.78% more dry matter accumulation at maturity stage, respectively. There was no significant difference in dry matter accumulation amount at heading stage between HY and MY. However, HY had markedly greater panicle dry matter accumulation from heading stage to maturity stage and, consequently, had markedly higher aboveground dry matter accumulation amounts relative to MY. Thus, HY is characterized by strong matter production after heading stage.

**Figure 6:**
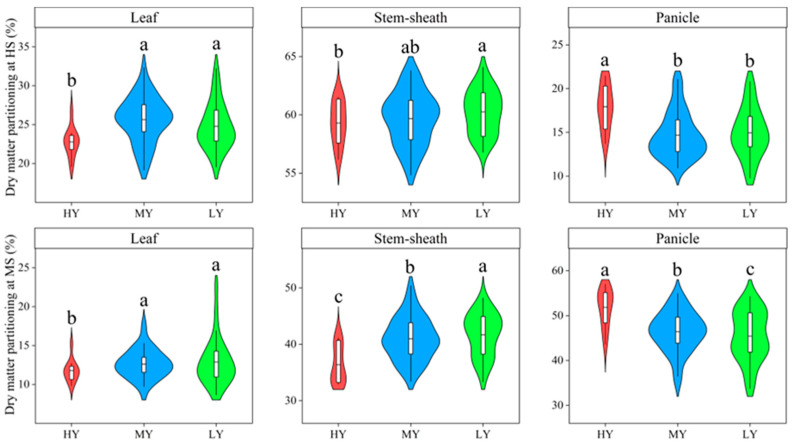
Dry matter partitioning of plant organs of different cultivars at heading stage and maturity stage. HS, heading stage; MS, maturity stage. Cultivars with significant differences (*p* < 0.05) are identified by different lowercase letters.

The proportion of dry matter partitioning to the leaf markedly decreased after the heading stage (Figure 6). In contrast, dry matter partitioning to the panicle significantly increased. Compared to MY and LY, HY had significantly decreased 10.92% and 9.69% dry matter partitioning to the leaf but increased 22.08% and 23.13% dry matter partitioning to the panicle at heading stage. The proportions of dry matter partitioning to the leaf, stem-sheath, and panicle significantly differed among cultivars at maturity stage. The dry matter of the stem-sheath of HY showed only minor differences in comparison to MY. However, the percentages of dry matter in the stem-sheath decreased. Both HY and MY produced greater quantities of panicle dry matter, by 50.49% and 20.46%, than LY. Additionally, the percentages of panicle dry matter were higher for HY by 15.34% and for MY by 2.93%.

**Figure 7:**
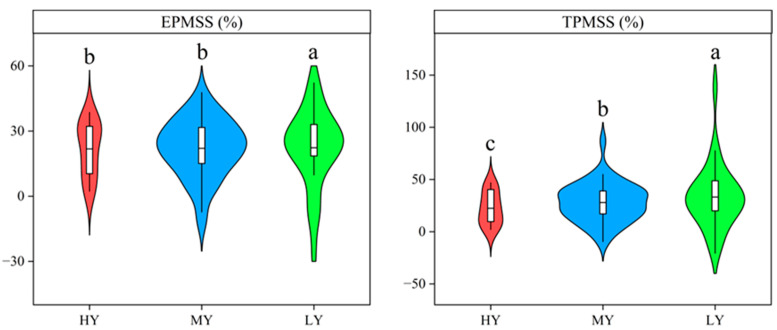
The export percentage of the matter in stem-sheath (EPMSS) and the transformation percentage of the matter in stem-sheath (TPMSS) from heading to the maturity stage of different cultivars. Cultivars with significant differences (*p* < 0.05) are identified by different lowercase letters.

Differences were observed amongst cultivars in both the percentage of exported matter and the percentage of matter transformed in the stem-sheath, as demonstrated in Figure 7. A reduction of 7.93% and 9.35% was recorded in the export ratio of matter in the stem-sheath of HY and MY cultivars, respectively, as compared to LY. Similarly, the transformation percentage of the matter in stem-sheath decreased with increasing grain yield. The transport ratio of biomass per stem-sheath of HY and MY decreased by 32.50% and 16.36% relative to LY, respectively.

### 2.4. LAI, SPAD, HI, and Grain–Leaf Ratio

**Figure 8:**
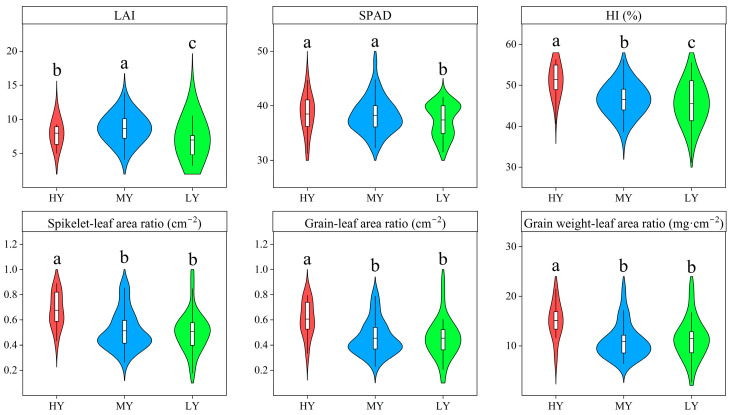
Source–sink relationship of different cultivars. Cultivars with significant differences (*p* < 0.05) are identified by different lowercase letters.

Cultivar type markedly affected the LAI, SPAD, and HI (Figure 8). Relative to LY, LAI, SPAD, and HI of HY and MY increased by 14.24% and 24.45%, 2.90% and 2.26%, and 12.24% and 1.57%, respectively. SPAD did not significantly differ between HY and MY. Compared with HY, the LAI of MY markedly increased 8.94%. However, the HI of MY significantly reduced compared to HY. Thus, canopy closure constrains dry matter production after heading stage, which resulted in a 9.51% decrease in HI over that of the HY. In addition, the grain–leaf ratio of HY significantly increased relative to MY or LY, which were responsible for high HI. These results indicated that HY had greater light utilization efficiency and large sink capacity.

### 2.5. Correlation Analysis

**Figure 9:**
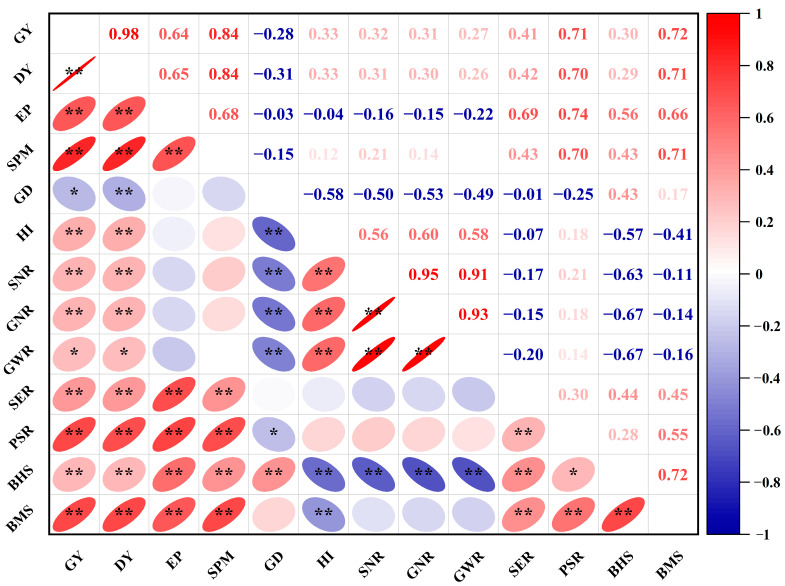
Relationships among grain yield and effective panicles, spikelets m^–2^, heading growth duration, seedling emergence rate, productive stem rate, source–sink relationship, and dry matter production characteristics. GY, grain yield; DY, daily yield; EP, effective panicles; SPM; spikelets per m^2^; GD, growth duration before heading; HI, harvest index; SNR, spikelet-leaf area ratio; GNR, grain-leaf area ratio; GWR, grain weight-leaf area ratio; SER, seedling emergence rate; PSR, productive stem rate; BHS, biomass at heading stage; BMS, biomass at maturity stage. *, *p* < 0.05; **, *p* < 0.01.

Grain yield and daily yield increased significantly with an increase in panicles per unit area, which showed a close correlation with both seedling emergence rate and productive stem rate (Figure 9). Grain yield and daily yield were significantly and positively correlated with the HI, SNR, GNR, GWR, SER, PSR, and biomass at heading stage and maturity stage. In contrast, there was a significant negative correlation between grain yield and growth duration from sowing to heading stage. Additionally, grain yield significantly increased with BHS and BMS.

### 2.6. Regression Analysis between Grain Yield and Growth Duration before Heading

**Figure 10:**
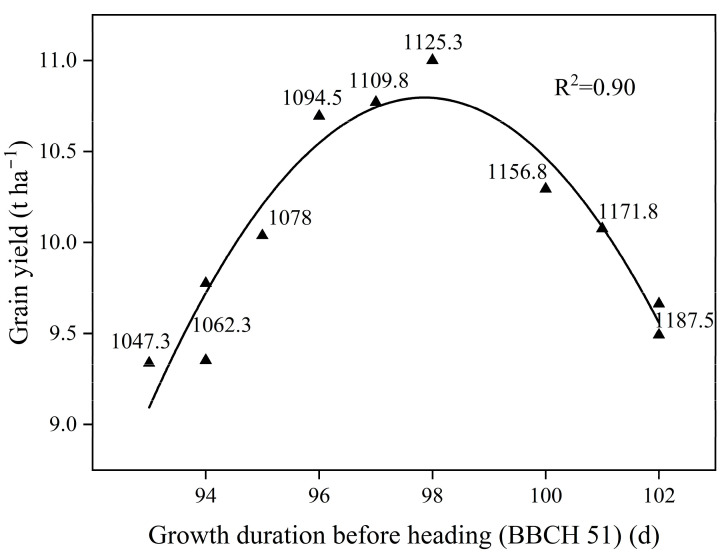
Relationship between grain yield and growth duration before heading (BBCH 51) for HY cultivars. Growing degree day (the base temperature as 10 °C) are indicated next to the symbols.

The regression model between grain yield and the period from seeding to beginning of heading (BBCH 51) (X) for high-yielding cultivars was established using the method of polynomial regression analysis (Figure 10), and the specific equation and correlation coefficient were as follows:

Y = −679.66 + 14.11X − 0.07X^2^, R^2^ = 0.901;

According to the regression equation, there was a single-peak curvilinear relationship between grain yield and growth duration before heading (BBCH 51) for HY cultivars. The grain yield first increased and then decreased with delaying the growth duration before heading (BBCH 51). Based on this quadratic equation, the maximum grain yield of rice cultivars suitable for dry direct seeding rice system may be obtained when the period from seeding to beginning of heading (BBCH 51) of rice cultivars were 98 d.

## 3. Discussion

Dry direct seeding rice is a promising option for rice production in China and other countries, based on less water resource input and higher production efficiency [11,34]. Currently, the cultivars being used in the dry direct seeding system are those bred for flooded, transplanted conditions. However, these cultivars are not well equipped with suitable traits for dry direct seeding system. Moreover, dry direct seeding system shortens the rice growth duration, so local transplanted rice cultivars cannot fully use the temperature and light resources, which might not achieve their potential yield in dry direct seeding system [19]. In this context, high-yielding rice cultivars with suitable traits and durations are urgently needed for dry direct seeding rice. Compared with MY and LY, the HY rice cultivar exhibits the characteristics of both high grain yield and shorter growth duration before heading (BBCH 51) (Table 1 and Figure 2), effectively meeting the requirements of dry direct seeding rice. Here, we analyzed the characteristics of yield components in dry direct seeding *japonica* inbred rice cultivars that have differing yield levels. Moreover, we identified certain selection criteria for high-yielding rice cultivars.

In this study, the grain yield of the tested rice cultivars varied from 5.75–11.00 t ha^−1^. We segregated these cultivars, based on the results of the hierarchical cluster analysis, into high-yielding (HY) cultivars, medium-yielding (MY) cultivars, and low-yielding (LY) cultivars. The significant difference in grain yield can be attributed to the variation in spikelets per unit area. (Table 1). The grain filling percentage or the 1000-grain weight was not responsible for the difference in yield. Previous studies have indicated that increasing the number of spikelets per panicle can enhance the yield of rice grains [7,35]. This study found that HY had a significantly higher yield than MY and LY because of a greater number of spikelets per panicle. It is widely acknowledged that there is a compensation between the number of panicles per unit area and the number of spikelets per panicle. However, HY had significantly higher effective panicles per unit area due to the increases in seedling emergence rate and productive stem rate relative to MY or LY (Figure 3), which suggests that enhancing the number of seedlings and productive stems alleviated the conflict between having a higher number of panicles per unit area and larger panicle size [36,37]. Moreover, our study showed that the effective panicles were significantly and positively associated with grain yield of dry direct seeding rice (Figure 9). Therefore, higher effective panicles and spikelets per panicle could be identified as main characteristics of HY *japonica* inbred rice cultivars.

The grain yield is the result of the accumulation of photosynthates in the organs of the plant [7]. It is imperative to improve dry matter production to increase yield potential. Our research findings indicate a significant positive correlation between grain yield, aboveground total dry weight, and relative dry matter accumulation from heading stage to maturity stage. Moreover, HY had markedly higher aboveground total dry weight at maturity stage (Figure 5). These findings corroborated the report of Zhou et al. [38], showing that grain yield increased with dry matter accumulation. From another perspective, grain yield is the product of the total aboveground biomass and harvest index. Our correlation analysis suggested that there was a significant positive correlation between grain yield and harvest index, and the harvest index of HY was significantly higher than that of MY and LY (Figure 8). It concurred with the results of Yang et al. [39] who found that a higher harvest index was conducive to the formation of higher grain yield. However, it is widely recognized that there is limited scope for further increasing the harvest index after the Green Revolution, and further improvement in rice yield might depend on increasing biomass production [40,41]. Thus, enhancing biomass accumulation seems to be the solution to progress in dry direct seeding rice yield potential based on a high harvest index.

Grain yield is also influenced by the allocation of dry matter after heading stage. Previous study reported that grain yield significantly increased with the proportion of dry matter partitioned to the stem-sheath at heading stage [42]. However, our results showed that the percentage of dry matter in the stem-sheath of HY was lower than that of MY and LY at heading stage and maturity stage (Figure 6). Moreover, HY and MY had lower matter output from stem plus sheath to the grain than that of LY (Figure 7). This was consistent with the findings of Yang et al. [43], who observed that low-yielding populations have a higher quantity of storage materials transported from the stem to the grain. Wu et al. [44] also found that, as the rice yield increases, the export ratio and the corresponding transport ratio of reserve substances per stem-sheath decrease significantly. Therefore, high-yielding varieties had the characteristics of lower export ratio of biomass per stem-sheath and the transport ratio of biomass per stem-sheath in the dry direct seeding system.

The formation of crop yield is a process of source–flow–sink interaction and is related to the production, transport, and accumulation of photosynthate [38,45]. Source organs and their productivity were the base for yield formation. In this study, HY and MY had stronger source supply capacity than LY because of greater LAI and SPAD. However, focusing only on high source supply capacity without improving the sink capacity will limit the accumulation of assimilates in grains. Previous studies have also shown that the supply of assimilates in dry direct seeding rice is often limited by sink capacity [30]. The grain–leaf ratio has been widely used as a comprehensive index for measuring the relationship between source and sink. Our study showed that the higher yield of dry direct seeding rice was attributed to improvements in sink size (spikelets number per m^2^) because HY had a higher grain–leaf ratio. Therefore, the rice population of HY cultivar showed a coordinated enhancement of sink size and source capacity.

Intercepted solar radiation during the growing season is a significant driver of crop canopy photosynthesis and biomass production [46,47]. The local transplanted rice cultivars cannot full use of light and temperature resources due to the shortening of the growth duration in the dry direct seeding system. Thus, rice cultivars with short growth durations are necessary for direct seeding system. In this study, there was a significant negative correlation between grain yield and growth duration before heading (BBCH 51), and the HY had a shorter period between seeding and beginning of heading (BBCH 51). Previous research has demonstrated that the growth duration of rice cultivars can be regulated by sowing date and pre-sowing seed treatment, which can improve the utilization of light and temperature resources [26,48]. Our study found that the growth duration before heading (BBCH 51) of HY cultivars suitable for the dry directed seeding rice system ranged from 93–102 days (Figure 10). Moreover, the results of regression analysis showed that the maximum yield could be obtained when the growth duration before heading (BBCH 51) of direct seeding rice cultivars was 98 days.

## 4. Materials and Methods

### 4.1. Experimental Sites and Soil Properties

Field trials were conducted in a farmer’s field located in Shenyang City, Liaoning Province, China (42°24′ N 122°59′ E), in 2020 and 2021. Shenyang has a temperate semi-humid continental climate. Between April and September 2020, the average ambient temperature and precipitation were 20.09 °C and 87.62 mm, respectively. Between April and September 2021, the average ambient temperature and precipitation were 19.69 °C and 119.16 mm, respectively. Before plowing, soil samples were collected from the assessed fields at a depth of 0–20 cm. The sample was air-dried, sieved, and analyzed in the laboratory using standard methods to estimate the physical and chemical properties. The experimental site soil texture was loam with a pH of 5.77, 14.45 g kg^−1^ of organic matter, 1.52 g kg^−1^ of total nitrogen, 107.42 mg kg^−1^ of available nitrogen, 15.84 mg kg^−1^ of available phosphorus, and 92.36 mg kg^−1^ of available potassium.

### 4.2. Tested Materials

Seventy-nine *japonica* inbred rice cultivars were used as experimental materials (Figure 1). These cultivars were broadly collected from Northeast China (Supplementary Table S1). Before sowing, the seeds were mechanically de-awned, and the unfilled seeds were separated by air. Meanwhile, the seed treated with azoxystrobin (Harbin Huolongshen Agricultural Biochemical Co., Ltd., Harbin, China) was thoroughly blended to ensure safe seedling emergence.

### 4.3. Experimental Design and Management

Field experiments were conducted with a completely random block design with three replications. The pretreated rice seeds were sown on 26 April 2020 and 28 April 2021, respectively. Fields were rotary tillage for 15 cm depth, suppression, then rotary tillage for 5 cm depth, harrowed, without peddling, and leveled before sowing. The size of each plot was 18 m^2^ (strip sowing, 9 m long, 2 m wide, and 10 rows with a 20 cm row spacing). The seeding rate was 90 kg hm^−2^ (390 seeds per square meter), and the sowing depth was 2 cm. The total N amounts of the N fertilizer application rate was 190 kg ha^−1^, which was sequentially applied as follows: 40% at basal, 30% at tillering stage, 20% at panicle initiation stage, and the remaining 10% at the heading stage of each cultivar. All nitrogen fertilizers were applied in the form of urea. Phosphorus (100 kg P_2_O_5_ ha^−1^ as triple superphosphate) was applied in all plots one day before sowing. Potassium (75 kg K_2_O ha^−1^ as potassium sulfate) were equal applied in all plots one day before sowing and at panicle initiation stage, respectively.

Water management: All treatments were flooded after sowing to maintain the soil moisture level and then wetting–drying alternation irrigation was performed after seedling emergence. A flood water depth of 3–5 cm was maintained after the four-leaf stage. Water was then drained at the maximum tillering stage to reduce unproductive tillers and re-watered at the panicle initiation stage with a 3–5 cm layer of water until the heading stage. Wetting–drying alternation irrigation was performed during the grain filling stage, and the water was drained 10 days before maturity.

Weed management: 480 g/L Clomazone EC (252 g a.i. ha^−1^), 75% Halosulfuron-methyl GR (56.25 g a.i. ha^−1^), 25 g/L Penoxsulam SC (22.5 g a.i. ha^−1^), and 30% Cyhalofop-butyl SC (225 g a.i. ha^−1^). Herbicides were applied twice, one using 480 g/L Clomazone EC (252 g a.i. ha^−1^), and 75% Halosulfuron-methyl GR (56.25 g a.i. ha^−1^) within three days after sowing, the other at six-leaf age using 25 g/L Penoxsulam SC (22.5 g a.i. ha^−1^) and 30% Cyhalofop-butyl SC (225 g a.i. ha^−1^). All herbicides were sprayed manually with a 3 WBD-18 knapsack electric sprayer. The sprayer was cleaned repeatedly before and after each application. After the second herbicide application for one day, a 3–5 cm water layer was maintained in the field for 5 days, and, during this period, the water layer was lower than 2/3 of the stems and leaves of rice.

### 4.4. Measurements and Sampling

#### 4.4.1. Growth Duration, Seedling Emergence Rate, Number of Stems, and Productive Stem Rate

The dates of sowing, heading stage, and maturity were recorded to determine the growth duration (GD). The seedling emergence rate was investigated 21 days after direct seeding. An area of 0.5 m^2^ was randomly selected three times in each plot to calculate the mean number of stems or panicles at the seedling emergence stage, maximum tillering stage, heading stage, and maturity stage. The seedling emergence rate (SER) and productive stem rate (PSR) were calculated as follows:

SER (%) = number of seedlings emerged/number of seeds sown × 100 (1)

PSR (%) = (number of panicles − number of seedlings)/(number of stems at maximum tillering stage − number of seedlings) × 100 (2)

#### 4.4.2. Dry Matter Accumulation and Allocation

At the heading and maturity stages, plants with an area of 0.5 m^2^ from each plot were selected for sampling. Following the recording of the number of panicles, the plant samples were separated into leaves, stems (culm plus sheath), and panicles. The dry weight of each organ was determined after oven-drying at 80 °C to achieve a constant weight. The total biomass was defined as the sum of the dry matter weight of the organs. The biomass at heading stage (BHS) and biomass at maturity stage (BMS) were used to represent the total biomass of each stage, respectively. The percentage of dry matter accumulations in the organs were calculated. The export percentage of the matter in stem-sheath (EPMSS) and the transformation percentage of the matter in stem-sheath (TPMSS) were measured as follows:

EPMSS (%) = (dry weight of stem-sheath at heading stage − dry weight of stem-sheath at maturity stage)/dry weight of stem-sheath at heading stage(3)

TPMSS (%) = (dry weight of stem-sheath at heading stage − dry weight of stem-sheath at maturity stage)/dry weight of panicle at maturity stage(4)

#### 4.4.3. Leaf Area Index, SPAD, and Grain–Leaf Ratio

During the heading stage, a leaf area meter (LI-3000, Li-Cor Inc., Lincoln, NE, USA) was used to measure the green leaf area of the plants and determine the leaf area index (LAI). The SPAD measurements were conducted using the SPAD-502 Chlorophyll Meter (Minolta Co. Ltd., Osaka, Japan). To present the source–sink relationship of rice, we used the grain–leaf area ratio, including three forms, spikelet-leaf area ratio (SNR), grain–leaf area ratio (GNR), and grain weight–leaf area ratio (GWR), which were calculated as follows:

SNR (cm^−2^) = total spikelets/leaf area at heading stage (5)

GNR (cm^−2^) = total grains/leaf area at heading stage (6)

GWR (mg cm^−2^) = grain yield/leaf area at heading stage (7)

#### 4.4.4. Grain Yield, Daily Yield, and Yield Components

To gather data on rice yield and yield components, plants were sampled from a 0.5 m^2^ area at maturity. Each sample was counted for panicle number to determine the effective panicles (EP) per square meter, and the plants were then separated into straw and panicles. The grains were manually separated from the straw by threshing the plants. The grain samples, which had been threshed, were air-dried and then submerged in tap water to separate filled and unfilled spikelets. They were then oven-dried at 80 °C to a constant weight. Three subsamples, each containing 20 g of filled grains, were manually counted to determine the 1000-grain weight. First, we divided 20 by the number of counted filled spikelets and then multiplied the result by 1000. Next, we calculated the number of filled spikelets by dividing the total dry weight of filled grains by the grain weight. We manually counted all of the unfilled spikelets and calculated the total number of spikelets per square meter (SPM) including both filled and unfilled ones. We calculated the spikelets per panicle by taking the ratio of the total spikelet number to the panicle number. Additionally, the seed-setting rate was calculated by taking the ratio of the filled spikelet number to the total spikelet number [38]. The grain yield at maturity, with a moisture content of around 20%, was estimated via manual harvesting of a 5 m^2^ (the middle five rows by 5 m) area of each plot with a sickle. The grain yields (GY) were converted to a moisture content of 14.5%. The daily yield (DY) was the ratio between grain yield per unit area to the whole duration days. The harvest index (HI) was the ratio between gain yield to aboveground biomass.

### 4.5. Statistical Analysis

The calculated daily yield of each of the 79 cultivars was used to classify them into different categories using Wards’ method of hierarchical cluster analysis. This analysis was conducted through the usage of the R (R 4.2.2) ‘stats’ package and ‘ggtree’ package. We used the least significant difference (LSD) test at a 0.05 probability level to compare the differences between treatments or multiple comparisons, using SPSS v. 26 (IBM Corp., Armonk, NY, USA). We used the Pearson correlation coefficient to analyze the relationship between various traits, at either the 0.05 or 0.01 probability levels. We used Origin version 2022 (OriginLab, Northampton, MA, USA) for the graphical illustration of the data. To establish the regression models between grain yield and growth duration of the heading stage, we used polynomial regression analysis.

## 5. Conclusions

Ensuring food security and high production efficiency are important prerequisites for achieving the transformation of rice cultivation system. In this study, 79 tested *japonica* inbred rice cultivars were classified into high-yielding (HY), medium-yielding (MY), and low-yielding (LY) cultivar. The HY had greater aboveground biomass and harvest index, mainly due to higher dry matter accumulation after heading stage. HY also increased the post-heading stage dry matter production and the accumulation of dry matter stored in the grains. In addition, the HY significantly enhanced both seedling emergency rate and productive stem rate, aiding in the conflict reduction between larger panicle size and higher panicle number per unit area. Furthermore, the HY cultivars coordinated the source and sink relationship by markedly improving grain–leaf ratio. Thus, HY enhanced the amounts of spikelets per unit area, leading to a 29.74–74.85% higher grain yield compared to MY and LY. Thus, rice cultivars with comparatively greater seedling emergency rate, productive stem rate, larger panicles, higher dry matter accumulation after heading stage, and higher apportionment of total dry matter towards grains at maturity stage can be chosen as HY *japonica* inbred rice cultivars for direct seeding cultivation.

## Figures and Tables

**Table 1:** Grain yield, daily yield, and yield components of different cultivars.

Cultivar	Grain Yield (t ha^−1^)	Daily Yield (kg ha^−1^ d^−1^)	Effective Panicles m^−2^	Spikelets per Panicle	Spikelets m^−2^ (×10^3^)	Grain Filling Rate (%)	1000-Grain Weight (g)
HY	10.05 a	64.03 a	458.03 a	111.00 a	49.98 a	89.05 a	24.88 a
MY	7.74 b	49.63 b	414.63 b	101.52 b	41.18 b	88.55 a	24.06 b
LY	5.75 c	36.81 c	307.16 c	101.84 b	30.85 c	88.09 a	25.16 a
Mean	7.38	47.25	383.95	102.95	38.87	88.46	24.55

HY, high-yielding cultivars; MY, medium-yielding cultivars; LY, low-yielding cultivars. Different lowercase letters within a column indicate significant (*p* < 0.05) differences among cultivars.

## Data Availability

The data used to support the findings of this study can be made available by the corresponding author upon request.

## Supplementary Materials

The following supporting information can be downloaded at: https://www.mdpi.com/article/10.3390/plants12193496/s1, Table S1: 79 rice cultivars collected from Northeast China.
