# Supplementary material for: Selection and Yield Formation Characteristics of Dry Direct Seeding Rice in Northeast China
Source: Plants (Basel). 2023 Oct 7;12(19):3496. doi: 10.3390/plants12193496 (PMC10575160; doi:10.3390/plants12193496)
Supplement: Supplementary file 1 [file plants-12-03496-s001.zip › plants-2593904-supplementary.pdf]

Table S1. 79 rice cultivars collected from Northeast China.

No.	Cultivar	Type	Donor
1	Shendao334	LY	Shenyang Agricultural University
2	Jinongda538	MY	Jilin Agricultural University
3	Jinongda823	LY	Jilin Agricultural University
4	Tonggeng797	LY	Tonghua Academy of Agricultural Sciences
5	Shendao333	LY	Shenyang Agricultural University
6	Shendao702	LY	Shenyang Agricultural University
7	Shendao14	LY	Shenyang Agricultural University
8	Shendao7	LY	Shenyang Agricultural University
9	Shennong315	LY	Shenyang Agricultural University
10	Shendao72	MY	Shenyang Agricultural University
11	Tongyu256	MY	Tonghua Academy of Agricultural Sciences
12	Tongyu838	LY	Tonghua Academy of Agricultural Sciences
13	Tonghe885	MY	Tonghua Academy of Agricultural Sciences
14	Xingchen899	MY	Jilin Xingchen seed Co., Ltd.
15	Jiyang108	LY	Jiyang Agricultural Science Research Institute
16	Jiyang100	LY	Jiyang Agricultural Science Research Institute
17	Tongke29	MY	Tonghua Academy of Agricultural Sciences
18	Hangeng8	MY	Jilin West Oasis Investment Co., Ltd.
19	Tonggeng890	LY	Tonghua Academy of Agricultural Sciences
20	Tonghe832	MY	Tonghua Academy of Agricultural Sciences
21	Shendao47	HY	Shenyang Agricultural University
22	Beigeng2	MY	Shenyang Agricultural University
23	Qiuguang	MY	Jilin Fengyou Agricultural Research Institute
24	Shendao11	MY	Shenyang Agricultural University
25	Tiegeng15	LY	Tieling Academy of Agricultural Sciences
26	Shendao240	HY	Shenyang Agricultural University
27	Shendao215	HY	Shenyang Agricultural University
28	Shendao88	HY	Shenyang Agricultural University
29	Jinongda738	HY	Jilin Agricultural University
30	Jida818	MY	College of Plant Science, Jilin University
31	Jida898	HY	College of Plant Science, Jilin University
32	Tongxi935	HY	Tonghua Academy of Agricultural Sciences
33	Tongke37	LY	Tonghua Academy of Agricultural Sciences
34	Songliao186	HY	Songliao Agricultural Science Research Institute
35	Jida618	HY	College of Plant Science, Jilin University
36	Songgeng16	HY	Wuchang Rice Research Institute of Heilongjiang Academy of Agricultural Sciences
37	Shendao18	MY	Shenyang Agricultural University
38	Shendao526	MY	Shenyang Agricultural University
39	Jigeng49	LY	Jilin Academy of Agricultural Sciences
40	Shendao536	LY	Shenyang Agricultural University
41	Shendao49	MY	Shenyang Agricultural University
42	Shendao69	LY	Shenyang Agricultural University
43	Shennong-dao546	MY	Shenyang Agricultural University
44	Tonggeng666	MY	Tonghua Academy of Agricultural Sciences
45	Jihong9	LY	Jilin Hongye seed Co., Ltd.
46	Songgeng29	MY	Wuchang Rice Research Institute of Heilongjiang Academy of Agricultural Sciences
47	Yangeng27	LY	Yanbian Academy of Agricultural Sciences

48	Shendao529	LY	Shenyang Agricultural University
49	Shendao316	LY	Shenyang Agricultural University
50	Tongxi937	HY	Tonghua Academy of Agricultural Sciences
51	Shendao223	MY	Shenyang Agricultural University
52	Shendao523	MY	Shenyang Agricultural University
53	Shendao520	MY	Shenyang Agricultural University
54	Shendao528	MY	Shenyang Agricultural University
55	Shendao357	LY	Shenyang Agricultural University
56	Jinongda859	MY	Jilin Agricultural University
57	Shendao531	LY	Shenyang Agricultural University
58	Shendao534	MY	Shenyang Agricultural University
59	Fuyu333	LY	Jilin West Oasis Investment Co., Ltd.
60	Kaigeng2	LY	Kaiyuan Agricultural Science Research Institute
61	Zhongke804	MY	Institute of Genetics and Developmental Biology, Chinese Academy of Sciences
62	Fengmin2000	MY	Liaoning Fengmin Agricultural High-tech Co., Ltd.
63	Jigeng836	MY	Jilin Academy of Agricultural Sciences
64	Futian2100	MY	Shenyang Futian seed Technology Co., Ltd.
65	Fuxingdao39	MY	Kaiyuan Haoshoucheng crop research institute
66	Zhongyan15	MY	Jilin Zhongyan Agricultural Development Co., Ltd.
67	Fuxing2838	MY	Kaiyuan Haoshoucheng crop research institute
68	Fuxing90	MY	Kaiyuan Haoshoucheng crop research institute
69	Suiyan6	MY	Heilongjiang Suiyan seed Industry Co., Ltd.
70	Tonghe837	LY	Tonghua Academy of Agricultural Sciences
71	Tongxi945	MY	Tonghua Academy of Agricultural Sciences
72	Tong-yuan568	LY	Tonghua Academy of Agricultural Sciences
73	Jiudao48	MY	Rice Research Institute of Jilin Academy of Agricultural Sciences
74	Tiegeng4	MY	Tieling Academy of Agricultural Sciences
75	Qinglin598	MY	Jilin Fengyou Agricultural Research Institute
76	Tiegeng11	MY	Tieling Academy of Agricultural Sciences
77	Jihong6	MY	Jilin Hongye seed Co., Ltd.
78	Jigeng528	MY	Jilin Academy of Agricultural Sciences
79	Songgeng22	MY	Wuchang Rice Research Institute of Heilongjiang Academy of Agricultural Sciences
